# Menstrual cycle length: a surrogate measure of reproductive health capable of improving the accuracy of biochemical/sonographical ovarian reserve test in estimating the reproductive chances of women referred to ART

**DOI:** 10.1186/s12958-015-0024-1

**Published:** 2015-04-10

**Authors:** Salvatore Gizzo, Alessandra Andrisani, Marco Noventa, Michela Quaranta, Federica Esposito, Decio Armanini, Michele Gangemi, Giovanni B Nardelli, Pietro Litta, Donato D’Antona, Guido Ambrosini

**Affiliations:** Department of Woman and Child Health, University of Padua, Gynecologic and Obstetric Clinic, Giustiniani 3 street, 35128 Padua, Italy; Department of Obstetrics and Gynaecology, University of Verona, Piazzale Ludovico Scuro 10 street, 37134 Verona, Italy; Department of Medicine-Endocrinology, University of Padua, Giustiniani 2 street, 35128 Padua, Italy

**Keywords:** Menstrual cycle length, Estimation of reproductive chances, Ovarian sensitivity index, Ovarian reserve, Ovarian response, Fertilization rate, Pregnancy rate, Infertility

## Abstract

**Background:**

Aim of the study was to investigate whether menstrual cycle length may be considered as a surrogate measure of reproductive health, improving the accuracy of biochemical/sonographical ovarian reserve test in estimating the reproductive chances of women referred to ART.

**Methods:**

A retrospective-observational-study in Padua’ public tertiary level Centre was conducted. A total of 455 normo-ovulatory infertile women scheduled for their first fresh non-donor IVF/ICSI treatment. The mean menstrual cycle length (MCL) during the preceding 6 months was calculated by physicians on the basis of information contained in our electronic database (first day of menstrual cycle collected every month by telephonic communication by single patients). We evaluated the relations between MCL, ovarian response to stimulation protocol, oocytes fertilization ratio, ovarian sensitivity index (OSI) and pregnancy rate in different cohorts of patients according to the class of age and the estimated ovarian reserve.

**Results:**

In women younger than 35 years, MCL over 31 days may be associated with an increased risk of OHSS and with a good OSI. In women older than 35 years, and particularly than 40 years, MCL shortening may be considered as a marker of ovarian aging and may be associated with poor ovarian response, low OSI and reduced fertilization rate. When AMH serum value is lower than 1.1 ng/ml in patients older than 40 years, MCL may help Clinicians discriminate real from expected poor responders. Considering the pool of normoresponders, MCL was not correlated with pregnancy rate while a positive association was found with patients’ age.

**Conclusions:**

MCL diary is more predictive than chronological age in estimating ovarian biological age and response to COH and it is more predictive than AMH in discriminating expected from real poor responders. In women older than 35 years MCL shortening may be considered as a marker of ovarian aging while chronological age remains most accurate parameter in predicting pregnancy.

## Background

Epidemiological data clearly demonstrated that, within and among women, menstrual cycles vary in length and regularity and that often a correlation exists between menstrual cycle characteristics and a variety of host, behavioural, occupational, and environmental factors [1]. However, few studies systematically examined whether menstrual cycle features are related to the most direct measures of reproductive health, fertility, and pregnancy outcome. The menstrual pattern is commonly understood to be fairly persistent within the individual, until the late 40s, when cycles lengthen before menopause. However, subtle gradual shortening of menstrual cycles occurs in the late 30s in parallel with the increase of FSH serum levels and the decrease of inhibin [2]. A shorter MCL may thus indicate a more advanced ovarian aging, which may proceed at a pace other than that of chronological aging [1,3,4]. That is, although ovarian aging is an inevitable process in all women, ovarian reserve differs significantly between individuals of similar age.

In developed Countries, the amount of women bearing children during the third and fourth decades of life is increasing [3]. In this view, it is not difficult to remark that many couples, experiencing age-related infertility due to diminished ovarian reserve (OR), turn to assisted reproduction techniques (ARTs).

In recent years, Clinicians involved in ARTs began focusing their attention and expending resources in defining “pretreatment estimation chances” and “individualization of treatment” in order to offer each woman the best treatment customized to her unique characteristics [5].

Although the personalization of IVF treatment may lead to improved patient compliance and clinical outcomes, it is far from easy, and largely depends on the accuracy of OR tests, usually performed by associating biochemical anti-mullerian hormone (AMH) assay and antral follicle count (AFC) [3,5-7]. The high accuracy of both AFC and AMH in estimating biological ovarian age has lead Clinicians to use them (in association with basal FSH and chronological age) to define the gonadotropin starting dose, to estimate the ART success and to optimize costs through an improvement in ovarian sensitivity index [8]. Unfortunately, a level of uncertainty remains concerning the AMH assay as differences between laboratory measurements persists. In addition, the absence of both a standardized measuring unit and a specific cut-off further contributes to reducing its accuracy, particularly when the values are located at the extremes of the Gaussian [9].

Women with extremely high or low AHM values are usually defined as expected high or poor responders and represent the category of patients in which cycle cancellation rate is highest due to OHSS or absence of ovarian response [5]. However, the overall accuracy of AFC in these two cohorts (women estimated high or poor responders) is lower than that of the general population and often does not improve the AMH accuracy [10,11]. Recent evidences demonstrated that long MCLs are associated with a greater number of antral follicle waves and higher ovarian response to hormonal stimulation (this seems to indicate higher reserve of primordial follicles in the ovary) [12]. On the contrary, short MCLs are associated with poor response to ovarian hyperstimulation, a markers of ovarian aging [13]. In consideration of the above, it seems intuitive that the menstrual diary should be routinely considered as a useful and low cost tool in estimating chances and improving reproductive outcome of ART cycles, particularly when patient report any previous ART cycle and their biological and chronological age do not match [3,14].

Aim of the study was to investigate whether menstrual cycle length may be considered as a surrogate measure of reproductive health, improving the accuracy of biochemical/sonographical ovarian reserve test in estimating the reproductive chances of women referred to ART.

## Methods

We conducted a retrospective observational study on normo-ovulatory infertile women scheduled for their first fresh non-donor IVF/ICSI treatment at Assisted Reproduction Unit of Gynecology and Obstetrics Clinic - Department of Woman and Child Health- University of Padua, between January 2011 and March 2014.

Our Study was defined exempt from IRB after consultation with the local ethical committee (“Comitato Etico per la Sperimentazione – Azienda Ospedaliera di Padova”). Approval from the local institutional review board for health sciences is not required for observational retrospective studies in which clinical management is not modified by the investigators. As usual in our unit, at admission all patients gave written informed consent for the use of their data in respect to the privacy law (Italian Law 675/96). Signed informed consent was obtained from each participant of this study.

We considered as eligible for the study women affected by infertility, aged between 18 and 50 years, with BMI ranging between 18 and 25, owning a personal menstrual diary of the six months preceding the ART treatment.

MCL was defined as number of days between the first day of bleeding until the day before the next bleeding period. The mean MCL during the preceding 6 months was calculated by physicians based on information regarding the beginning of menstrual cycle for all months considered contained in and electronic database (collected every month by telephonic communication by single patients).

Cycles were excluded from the calculation if they were subject to any kind of hormonal intervention. Patients were excluded from the study if a reliable menstrual cycle history could not be obtained.

We excluded patients with history of smoking in the previous 12 months, deep endometriosis with elevated CA125 serum value [15], abnormalities in karyotype, mutations of the cystic fibrosis gene, acquired or inherited thrombophilia and immunological disorders, previous chemo and/or radio therapy for neoplasia, untreated uterine diseases (such as endometrial polyps, sub mucous myomas, intrauterine synechiae and/or uterine septus) [16,17]. We also excluded patients who received low-dose aspirin during treatment [18] and patients with personal history of diabetes and thyroid disorders in order to avoid a possible bias in evaluating the pregnancy rate [19,20].

### Intervention

All patients were subjected to pre-treatment basal ovarian reserve test by biochemical assays of FSH and AMH levels in association with sonographic AFC [3].

According to pre-treatment ovarian reserve assessment, all patients received the most adequate stimulation protocol (long agonist protocol and variable-scheme short antagonist protocol) according to our Units Protocol. In detail all patients were treated by rFSH and hMG (alone or in combination) for ovarian stimulation, using a starting dose, maintained for the first 5 days, of 100 IU, 225 IU and 300 IU day in estimated high, normal and poor responders, respectively. All women performed subcutaneous injection of 250 mg rhCG for ovulation induction (when there were 2 or more follicles ≥16 mm in diameter with accompanying follicles ≥12 mm and an adequate E2 response) and after 36 hours underwent oocyte retrieval. Embryo transfer (ET) was performed 3 days after pick-up with transfer of 3 embryo, when obtained, after selection for quality [21]. All patients received high dose progesterone supplementation (600 mg vaginally and 100 mg intramuscular for day) for luteal phase support until β-hCG assay was performed 14 days after ET [22]. Clinical pregnancy was confirmed by ultrasonographic visualization of one or more gestational sacs or definitive clinical signs of pregnancy and ongoing pregnancy in the event of an uncomplicated pregnancy over 12 gestational weeks.

### Data collection

For all women we collected data regarding: age, BMI, ovarian reserve test (b-FSH, b-AMH, b-AFC), mean length of menstrual cycle in the six months prior to ART cycle, total dose of gonadotropins administered (IU), E2 at ovulation induction (nmol/L), endometrial thickness at pick-up (mm), total number of oocytes, number of MII oocytes, MII oocytes fertilization ratio (considering 6 as the max number of oocytes fertilized, in respect to Italian Law), number of obtained embryos, clinical, ongoing pregnancy and miscarriage rate.

We considered AGE_class_1 patients older than 40 years, AGE_class_2 patients aged between 35–40 years, AGE_class_3 patients aged between 26–34 years and AGE_class_4 patients younger than 26 years.

We considered MCL_class_1 when the mean value of the menstrual cycle was higher than 31 days, MCL_class_2 when ranged between 30–31 days, MCL_class_3 when ranged between 28–29 days, MCL_class_4 when ranged between 26–27 days and MCL_class_5 when less than 26 days.

We considered AMH cohort_1 when the AMH assay showed a value ranging between 0.1-0.4 ng/ml, AMH cohort_2 when the value ranged between 0.5-1.1 ng/ml and AMH cohort_3 when the value was higher than 1.1 ng/ml.

### Objectives

Primary objective was to evaluate whether a correlation exists between the various classes of mean MCL and number of MII oocytes, MII oocytes fertilization ratio and OSI (oocytes recovered*1000/total dose of FSH) in any considered subgroup of analyses [23].

Secondary objective was to detect if patients defined as expected poor responders (subgroup of analyses) according to Bologna Criteria (older than 40 years, AFC 5–7 follicles and/or AMH 0.5–1.1 ng/ml) [24] showed different ovarian response (mean number of MII oocytes) for each cohort of AMH value in relation to the class of mean MCL.

Finally, in estimated “normo-responder” patients we assessed clinical pregnancy rate for fresh embryo transfer in the different classes of MCL according to their class of age.

### Statistical analysis

Statistical analysis was performed by SPSS software (Chicago,IL) for Windows version 19, applying parametric and non-parametric tests when appropriate. The Kolmogorov–Smirnov test was used to assess the normality of distribution. Continuous variables were expressed as absolute numbers, average ± standard deviation, and analyzed by Student-*t* test or Anova test when appropriate; categorical variables were expressed as percentages and analyzed through the *χ*2 test or the Fisher’s exact test, when appropriate. Statistical significance was defined as p values < 0.05.

## Results

In the considered time frame, we collected data pertaining to 455 eligible patients aged between 23 and 48 years (mean value 36.42 ± 7.25). Data regarding general features (BMI, bFSH, bAMH, bAFC, mean MCL), stratified by age, were reported in detail in Table 1.

**Table 1:** **General features of eligible patients stratified for class of age**

**VARIABLES**	**ALL PATIENTS [455] [MEAN(±STANDARD DEVIATION)]**	**AGE CLASS and PATIENT NUMBER**
		**[CLASS_1 (>40 years) 157]**
		**[CLASS_2 (35–40 years) 115]**
		**[CLASS_3 (26–34 years) 155]**
		**[CLASS_4 (<26 years) 28]**
		**[MEAN(±STANDARD DEVIATION)]**
*PATIENT’S AGE (years)*	[36.42 (7.25)]	CLASS_1 [44.3 (2.4)]
		CLASS_2 [37.9 (1.5)]
		CLASS_3 [29.4 (2.6)]
		CLASS_4 [24.4 (0.7)]
*BMI*	[22.1 (2.1)]	CLASS_1 [22.7 (1.6)]
		CLASS_2 [22.4 (2.1)]
		CLASS_3 [21.5 (2.4)]
		CLASS_4 [22.1 (2.1)]
*bFSH*	[9,8 (4.3)]	CLASS_1 [12.5 (4.3)]
		CLASS_2 [10.7 (4.3)]
		CLASS_3 [6.9 (1.8)]
		CLASS_4 [6.5 (2.0)]
*bAMH*	[2.0(1.7)]	CLASS_1 [0.7 (0.8)]
		CLASS_2 [1.7 (1.1)]
		CLASS_3 [3.3 (1.7)]
		CLASS_4 [3.6 (1.7)]
*bAFC*	[9.9(6.4)]	CLASS_1 [5.2 (3.3)]
		CLASS_2 [8.5 (3.9)]
		CLASS_3 [14.6 (5.9)]
		CLASS_4 [15.9 (7.7)]
*mean MCL*	[28.1(2.4)]	CLASS_1 [26.4 (1.9)]
		CLASS_2 [28.2 (1.7)]
		CLASS_3 [29.4 (2.1)]
		CLASS_4 [29.7 (2.7)]

According to patients’ age and ovarian reserve test, performed before the initiation of treatment, 185 patients (40.7%) were expected poor-responders, 212 patients (46.6%) normo-responders and 58 patients (12.7%) high-responders. Regarding MCL diary, 21 patients (4.6%) were classified as MCL_class_1, 73 patients (16.1%) as MCL_class_2, 128 patients (28.1%) as MCL_class_3, 146 patients (32.1%) as MCL_class_4 and 87 patients (19.1%) as MCL_class_5. Data about controlled ovarian hyperstimulation cycles (FSH total dose, E2 max at ovulation induction, endometrial thickness at pick-up, total number of retrieved oocytes, number of MII oocytes, oocytes fertilization ratio and OSI) were reported in detail in Table 2.

**Table 2:** **Data about ovarian controlled hyperstimulation cycles stratified for class of age**

**VARIABLES**	**ALL PATIENTS [MEAN(±STANDARD DEVIATION)]**	**AGE CLASS and PATIENT NUMBER**
		**[CLASS_1 (>40 years) 157]**
		**[CLASS_2 (35–40 years) 115]**
		**[CLASS_3 (26–34 years) 155]**
		**[CLASS_4 (<26 years) 28]**
		**[MEAN(±STANDARD DEVIATION)]**
*FSH total dose*	455 [2983.9(856.5)]	CLASS_1 [3596.8 (520.1)]
		CLASS_2 [3100.0 (511.1)]
		CLASS_3 [2405.3 (848.5)]
		CLASS_4 [2271.4 (933.6)]
*E2 max*	455 [5.7(3.2)]	CLASS_1 [3.4 (1.9)]
		CLASS_2 [5.1 (2.7)]
		CLASS_3 [8.1 (2.5)]
		CLASS_4 [8.1 (2.9)]
*EE max*	455 [10.9(2.1)]	CLASS_1 [9.7 (1.5)]
		CLASS_2 [10.6 (2.1)]
		CLASS_3 [11.9 (2.1)]
		CLASS_4 [12.3 (1.9)]
*Total oocytes*	455 [7.4 (4.7)]	CLASS_1 [3.9 (3.1)]
		CLASS_2 [6.6 (2.8)]
		CLASS_3 [10.9 (4.1)]
		CLASS_4 [11.4 (4.9)]
*MII oocytes*	448 [5.1 (3.3)]	CLASS_1 [2.5(2.3)]
		CLASS_2 [4.7 (1.6)]
		CLASS_3 [7.5(3.1)]
		CLASS_4 [7.7 (2.9)]
*OSI*	448 [3.5 (4.2)]	CLASS_1 [1.2 (1.2)]
		CLASS_2 [2.2 (1.1)]
		CLASS_3 [6.2 (5.4)]
		CLASS_4 [7.1 (5.6)]
*Fertilization ratio*	402 [0.74 (0.22)]	CLASS_1 [0.64 (0.34)]
		CLASS_2 [0.79 (0.15)]
		CLASS_3 [0.78 (0.08)]
		CLASS_4 [0.75 (0.52)]

Among the 455 patients, 53 failed to receive embryo-transfer for the following causes: in 7 patients (1.6%) no oocytes were collected, in 24 patients (5.3%) no MII oocytes were found, and in 22 patients (4.8%) no oocytes were fertilised. Concerning the remaining 402 patients (88.3%), 110 achieved a clinical pregnancy (27.4%) and, of these, 62 patients (56.4%) reported an ongoing pregnancy.

**Figure 1:**
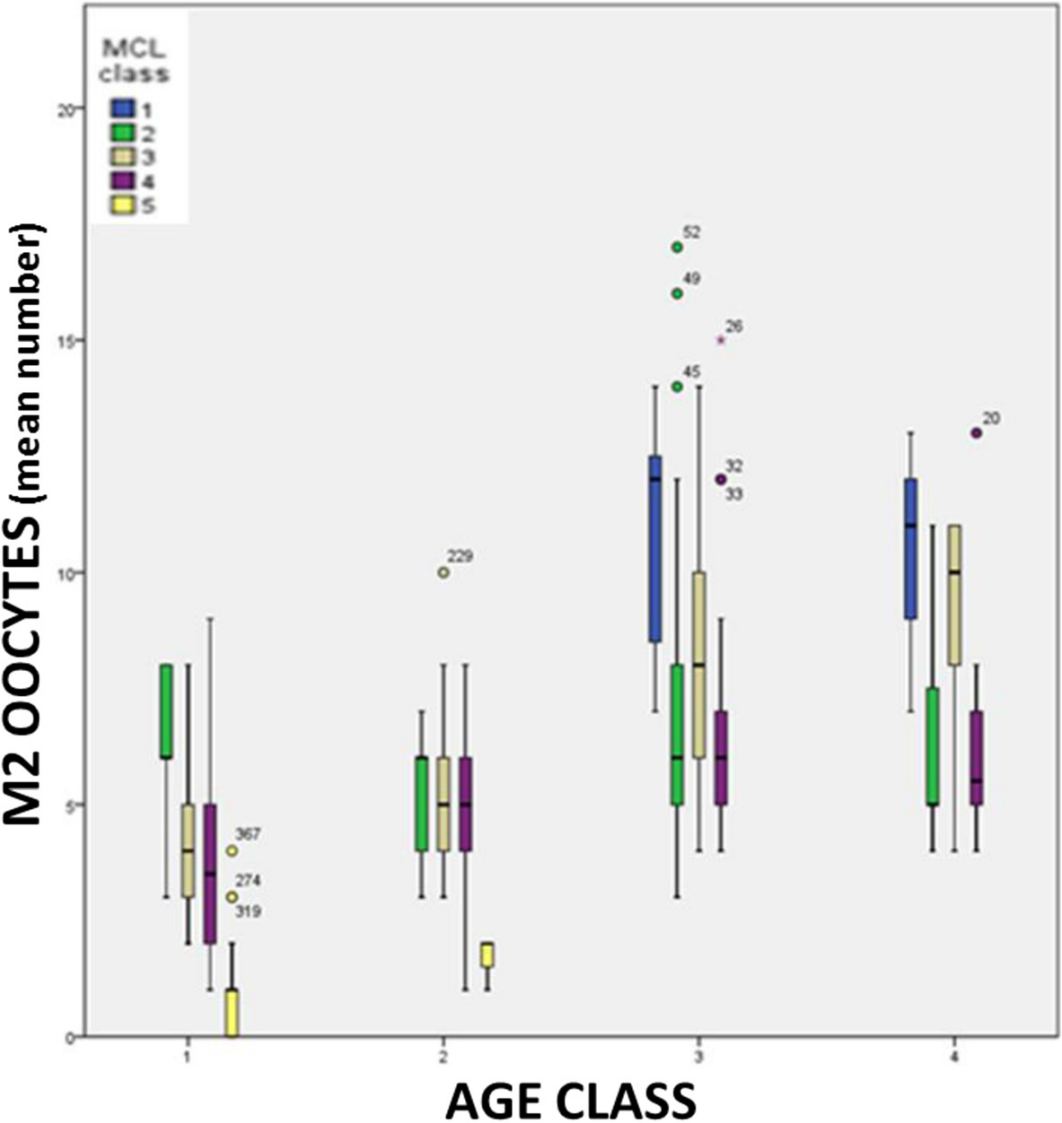
**The stratification of data according to patient’s class of age, mean MCL class and mean number of MII oocytes retrieved.** AGE_class_1: patients older than 40 years; AGE_class_2 patients aged between 35–40 years; AGE_class_3 patients aged between 26–34 years; AGE_class_4 patients younger than 26 years) (MCL_class_1: >31 days, MCL_ class_2: 30–31 days, MCL_ class_3: 28–29 days, MCL_ class_4: 26–27 days, MCL_ class_5: <26 days.

The stratification of data according to patient’s class of age, mean MCL class and mean number of retrieved MII oocytes showed that a statistically significant difference exists between different MCL classes in patients older than 40 years (mean MII oocytes: 6.1 in MCL_class_2, 4.6 in MCL_class_3, 4.3 in MCL_class_4 and 1.3 in MCL_class_5 respectively) ***[p < 0.001]***. Statistically significant differences were also found in patients belonging to MCL_class_1 as compared to those in MCL_class_2, MCL_class_3 and MCL_class_4 aged less than 35 years (in both Age_class_3 and 4) ***[p < 0.05]***. Within Age_class_2 only patients belonging to MCL_class_5 showed significant differences compared to the remaining MCL classes (1.9 versus 5.2, respectively) ***[p < 0.01]***. On the contrary, the comparison between MCL_class_2, 3 and 4 in Age_class_2, 3 and 4 did not show any statistically significant differences, despite better outcomes found in MCL_class_3 (Figure 1).

**Figure 2:**
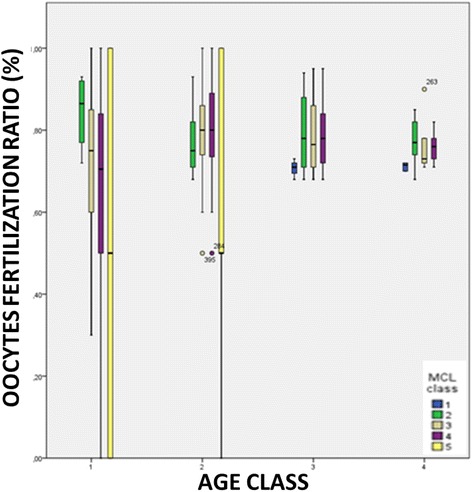
**The stratification of data according to patient’s class of age, mean MCL class and MII oocytes fertilization ratio.** AGE_class_1: patients older than 40 years; AGE_class_2 patients aged between 35–40 years; AGE_class_3 patients aged between 26–34 years; AGE_class_4 patients younger than 26 years) (MCL_class_1: >31 days, MCL_ class_2: 30–31 days, MCL_ class_3: 28–29 days, MCL_ class_4: 26–27 days, MCL_ class_5: <26 days.

The stratification of data according to patient’s class of age, mean MCL class and MII oocytes fertilization ratio showed that a statistically significant difference exist between the different MCL classes in patients older than 40 years (mean fertilization ratio: 86% in MCL_class_2, 77.5% in MCL_class_3, 71% in MCL_class_4 and 50.5% in MCL_class_5, respectively) ***[p < 0.01]***. Regarding patients aged between 35 and 40 years, statistical differences were found only between MCL_class_5 (50.8%) and MCL_class_2, 3 and 4 (mean value 79.2%) ***[p < 0.01]***. No differences were found when comparing different MCL classes in both Age_class_3 and 4 despite MCL_class_1 showed a lower fertilization rate than MCL_class_2, 3 and 4 (Figure 2).

**Figure 3:**
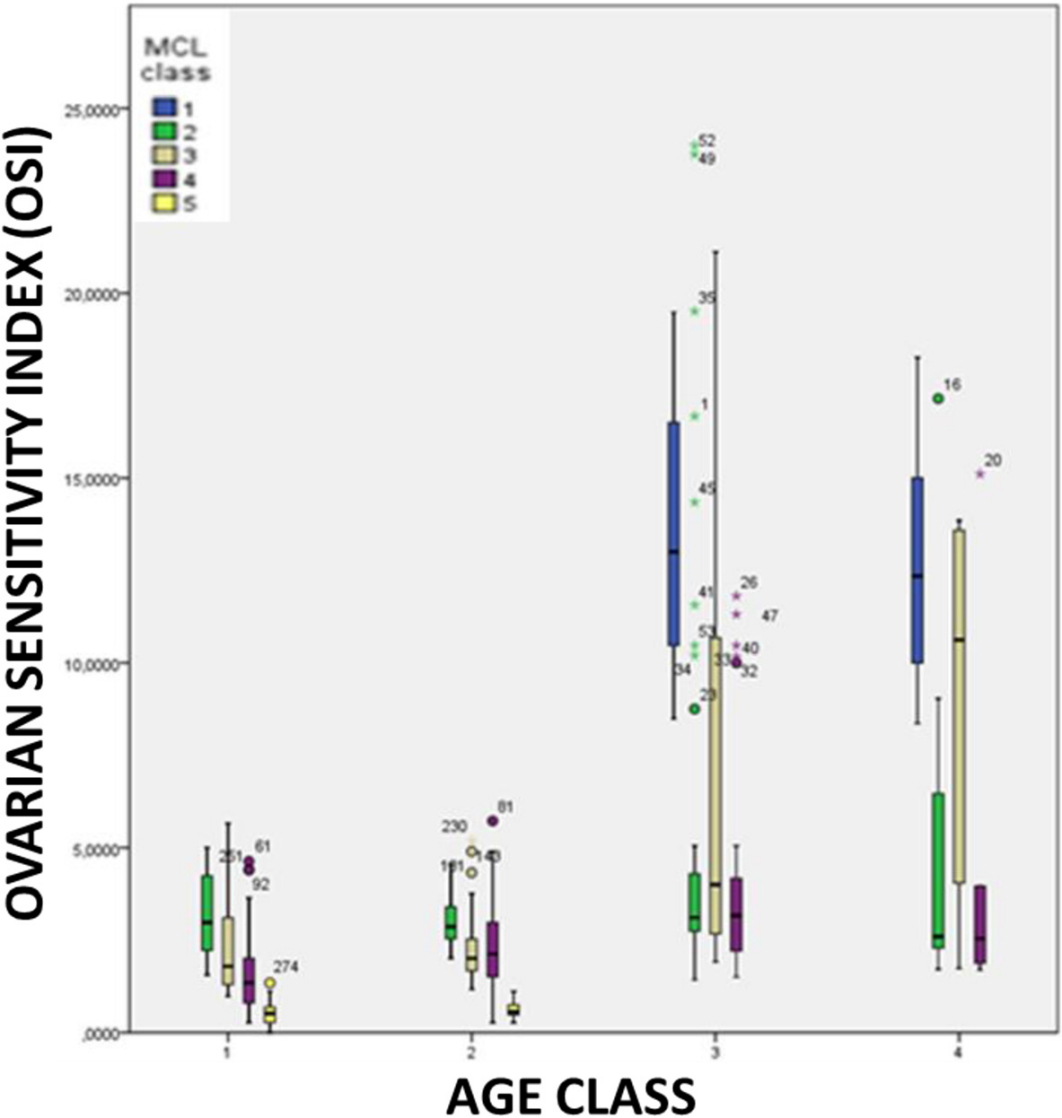
**The stratification of data according to patient’s class of age, mean MCL class and OSI.** AGE_class_1: patients older than 40 years; AGE_class_2 patients aged between 35–40 years; AGE_class_3 patients aged between 26–34 years; AGE_class_4 patients younger than 26 years) (MCL_class_1: >31 days, MCL_ class_2: 30–31 days, MCL_ class_3: 28–29 days, MCL_ class_4: 26–27 days, MCL_ class_5: <26 days.

The stratification of data according to patient’s class of age, mean MCL class and OSI showed that a statistically significant differences exists between MCL_class_1 and MCL_class_2, 3 and 4 in patients younger than 35 years (in both Age_class_3 and 4) ***[p < 0.001]***. Considering Age_class_3 and 4, MCL class_3 showed better OSI than MCL class_2 ad 4 ***[Age class_3 p < 0.05 and Age class_4 p < 0.001, respectively]***. Regarding Age_class_1 and 2, OSI differed significantly in relation to the different MCL classes, with a trend in reduction observed from MCL_class_2 to MCL_class_5 ***[p < 0.05]*** (Figure 3).

**Figure 4:**
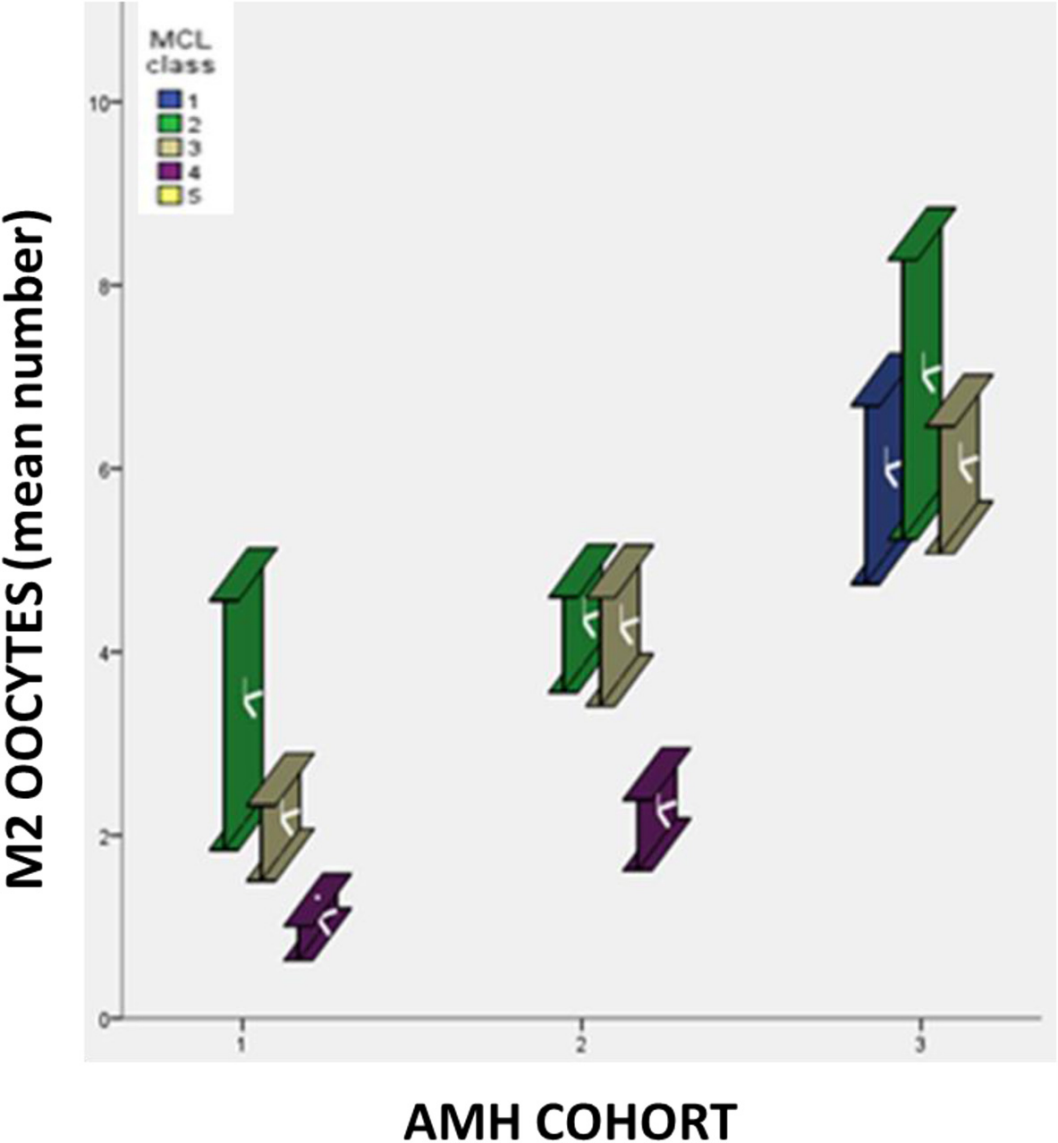
**The stratification of data according to MCL classes and AMH cohorts in relation to mean MII oocytes retrieved (considering the cohort of patients estimated poor-responders according to the Bologna Criteria).** AGE_class_1: patients older than 40 years; AGE_class_2 patients aged between 35–40 years; AGE_class_3 patients aged between 26–34 years; AGE_class_4 patients younger than 26 years) (MCL_class_1: >31 days, MCL_ class_2: 30–31 days, MCL_ class_3: 28–29 days, MCL_ class_4: 26–27 days, MCL_ class_5: <26 days.

Considering the cohort of patients defined as expected poor-responders according to the Bologna Criteria, the stratification of data according to MCL classes and AMH cohorts in relation to mean MII oocytes retrieved showed that no differences exist between MCL classes in AMH cohort_3. Instead, significant differences were found in AMH_cohort_2 between MCL_class_5 and MCL_class_3 and 4 ***[p < 0.01]***, as well as in AMH_cohort_1 a statistically significant worsening trend was observed when comparing MCL_class_2 versus MCL_class_3 versus MCL_class_4 ***[p < 0.05]*** (Figure 4).

**Figure 5:**
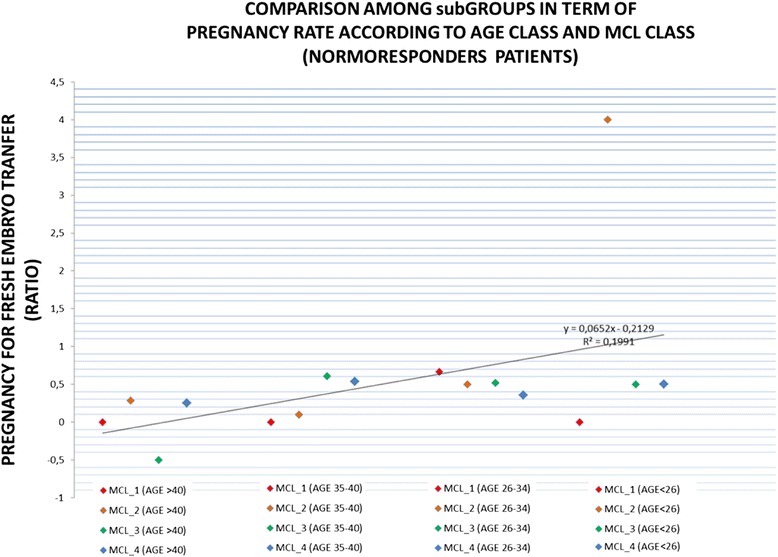
**The stratification of data according to MCL classes, Age classes and clinical pregnancy rate for fresh embryo transfer.** AGE_class_1: patients older than 40 years; AGE_class_2 patients aged between 35–40 years; AGE_class_3 patients aged between 26–34 years; AGE_class_4 patients younger than 26 years) (MCL_class_1: >31 days, MCL_ class_2: 30–31 days, MCL_ class_3: 28–29 days, MCL_ class_4: 26–27 days, MCL_ class_5: <26 days).

Finally, considering the cohort of patients estimated normo-responders, the stratification of data according to MCL classes, Age classes and clinical pregnancy rate for fresh embryo transfer showed that no differences exist between MCL classes and any Age class except for MCL_class_2 (better ratio) and MCL_class_1 (worst ratio) in very young patients (Age_class_4). On the contrary, as expected, a significant linear correlation was found between low women’s age and pregnancy ratio independent from the MCL classes ***[p < 0.05]*** (Figure 5).

## Discussion

For most women, the regularity of the menstrual cycle is characteristic of the major part of reproductive life. In the few years following menarche and those preceding menopause, many experience cycle irregularity. In fact, the onset of such irregularity in later years is widely regarded as the onset of the transition from regular cycles to the final menses, or menopause [25].

Decreasing follicle numbers, with a decreased number of fully functioning granulosa cells, initially causes a reduction in the secretion of inhibin B. The consequence of which is an increase in FSH secretion in late luteal and follicular phase, that may, in turn, lead to earlier initiation of follicle development and to a shortened follicular phase of continuing regular menstrual cycles. Intuitively, young women with high numbers of primordial follicles may frequently experience menstrual cycles longer than 30 days [25].

Despite the fact that certain epigenetic factors have been associated with abnormalities in menstrual cycle patterns (age, BMI, sedentary lifestyle, intense activity, alcohol consumption, smoking, caffeine consumption), aging alone is able to influence MCL in the absence of known risk factors taking into account interpersonal differences sometimes insignificant, sometimes significant [26].

There is accumulating evidence that menstrual cycle characteristics are the most direct measures of spontaneous reproductive health, fertility, and pregnancy outcome in the general population [1].

Thus, it may be expected that a large portion of women affected by subfertility/infertility and referring to ARTs may show MCL abnormalities due to anovulation, advanced age, premature ovarian failure and alteration in diet and BMI.

Considering that ovarian stimulation is an integral part of ARTs and, consequently, the responsiveness to gonadotropin stimulation is a factor which has important implications on treatment, pre-treatment prediction of ovarian response should be the most important parameter used to optimize treatment success and live birth rate and minimize risks and costs [8].

Countless efforts have been made to establish the best predictors of ovarian responsiveness, but the wide range of ovarian reserve tests proposed suggest that no single test provides a complete or sufficiently accurate estimate [10].

Very recently it has been internationally accepted that the combination of AFC with basal AMH assay represents a good tool to assess “biological” ovarian age, and therefore, to establish with high accuracy the gonadotropin starting dose and have a good predictability of ART success while increasing both OSI and pregnancy rate [5,6,8,9,11,24,27].

Disseldorp et al. in 2010 comparing the inter- and intra-cycle stability of AFC and AMH demonstrated that, despite AMH levels showed a positive correlation with AFC levels, AMH seems to have less individual intra- and inter-cycle variation than AFCs. Authors explained the higher stability of AMH measurements assuming that AMH levels are also determined by a cohort of pre-antral or small antral follicles, whereas the number of larger and visible antral follicles, expressed by the AFC, may be more prone to short-term variation. Possible explanations for a varying cohort of antral follicles might be cyclic differences in decay or growth rate which may depend on the presence of larger follicles in the early follicular phase [28].

Unfortunately, in clinical practice clinicians often observe different ovarian responses in patients with comparable values of serum AMH, particularly when the values are extremely low or high. Our intent, first in literature to our knowledge, was to determine whether menstrual cycles can act as a surrogate measure of reproductive health and may have a role in better defining the reproductive chances of women referred to ART.

Our data clearly demonstrated that, when AMH serum value is lower than 1.1 ng/ml and patients are estimated poor responders, the ovarian response to hyperstimulation is very different between individual patients and strongly correlates with MCL. Patients with a history of MCL shorter than 28 days showed a linear reduction of ovarian response in accordance with MCL shortening. In fact, in patients with AMH value less than 0.4 ng/ml (cohort 1) an average of 3.5 oocytes per cycle were collected in patients with a 30 day MCL length while only1.2 oocytes per cycle were retrieved in those with MCL length of 26 days.

In addition, our data showed that MCL correlated with the fertilization rate of the MII oocytes retrieved, particularly in patients older than 35 years. In fact, considering patients older than 40 years (estimated poor responder according to Bologna criteria) in which the number of MII oocytes collected is usually low, in patients with MCL of about 30 days a fertilization rate of almost 90% was observed compared to the 50% observed in those with MCL less than 26 days. Interestingly, this data, though it remains to be confirmed in a large scale population, leads to hypothesize that MCL may be considered as a parameter of oocyte quality superior to chronological age, as currently accepted [29].

However, patients’ age continues to maintain its leading role in assessing the chance of conceiving, since our data clearly demonstrated that clinical pregnancy was associated with age class more than MCL class. Probably MCL is a good mirror of “ovarian biological age” while “chronological age” is one of the most important factors affecting endometrial receptivity, probably the main factor limiting the success of ART in patients over 35 years [29].

Our study also demonstrated that in younger and expected normo-responder patients the MCL evaluation can bring benefits: data about OSI clearly demonstrated that even in patients aged between 26–34 years and in patients younger than 26 years MCL longer than 31 days was associated with an OSI of about 12.5 compared to an OSI of about 5 when MCL was shorter than 30 days.

Interestingly, but not fully explained by our data, in women younger than 35 years and with MCL ranging between 26 and 30 days (estimated as a regular period for general population) women with cycle length of 28 days showed better ovarian response and OSI than those with MCL between 26–28 and 28–30 days. Probably in patients without luteal phase abnormalities, the MCL deviating from 28 days (both in increasing and in decreasing duration) may be considered as an indicator of alteration in the follicular phase which may affect the success of both singular (spontaneous cycles) and multiple follicular recruitment (assisted cycles).

According to the results of our study and awaiting further validation, we strongly suggest to consider the introduction in routine clinical practice of MCL evaluation, in so much as we consider this parameter to be an indicator of ovarian age superior to that of FSH and chronological’ age. The large scale applicability and the good accuracy (for any biological and chronological age) of MCL in estimating ovarian response, OSI and fertilization rate allows Clinicians to consider it as an inexpensive good tool capable of improving the accuracy of biochemical/sonographical ovarian reserve test and to better estimate the ART success rate.

The strength of our data is due to the following: first IVF cycle for all patients, strict inclusion criteria, medical collection of menstrual diary for all months considered (not self-reported by patients), good number of patients for any class of “chronological” and “biological” age, single centre performance of AFC and ovarian stimulation protocol, single laboratory (and units measure) for measurement of AMH and other biochemical assays.

Unfortunately our study was not free of limitations: retrospective collection of data, variability in ovarian stimulation protocols (choosing the most appropriate for each patients), use of both recombinant and purified gonadotropins (alone or in combination), assessment of oocytes fertilization ratio using 6 oocytes as a maximum number to fertilize (according to the Italian law), lack of data regarding the cumulative pregnancy rate considering subsequent non-fresh gametes fertilization and embryo transfers, absence of data regarding bleeding patterns of single patients (qualitative and quantitative) may represent a potential bias affecting our results.

In consideration of our study limitations represented by the relatively small population size which was further divided in sub-groups, we recommend caution in evaluating our results. However the absence of cost and the large scale implementation of MCL estimation prior to initiating ART treatments strongly suggest validation by large-scale multicentre perspective trial.

## Conclusion

Menstrual cycles may be considered as a surrogate measure of reproductive health and may have a role in improving the accuracy of biochemical/sonographical ovarian reserve test in estimating the reproductive chances of women referred to ART.

In women younger than 35 years, MCL over 31 days may be associated with increased risk of OHSS and with a good OSI.

In women older than 35 years, and particularly than 40 years, MCL shortening may be considered a marker of ovarian aging and may be associated to poor ovarian response, low OSI and reduced fertilization rate.

When AMH serum value is lower than 1.1 ng/ml and patients older than 40 years, MCL may help Clinicians to discriminate real to those expected poor responders.

MCL is a good inexpensive tool to estimate ovarian age and response to hyperstimulation protocols, but the chronological age remains most accurate in predicting clinical pregnancy.

## Abbreviations

AFC: Antral follicle count
AMH: Anti mullerian hormone
ART: Assisted reproductive technique
BMI: Body mass index
E2: 17β estradiol
hMG: Human menotropin
IVF: In-vitro fertilization
MCL: Menstrual cycle length
MII: Metaphase 2 (oocytes)
OHSS: Ovarian hyper stimulation syndrome
OR: Ovarian reserve
OSI: Ovarian sensitivity index
rFSH: Recombinant follicle stimulant hormone
rhCG: Recombinant human chorionic gonadotropin
